# Natural Advantages of Preparation of Composites from Minerals: Effect of Bauxite Addition on the Microstructures and Properties of Fe-Al_2_O_3_ Based Composites

**DOI:** 10.3390/ma12091456

**Published:** 2019-05-06

**Authors:** Yuxin Chen, Baowei Li, Yu Shi, Shunli Ouyang

**Affiliations:** 1 Key Laboratory of Integrated Exploitation of Bayan Obo Multi-Metal Resources, Inner Mongolia University of Science and Technology, Baotou 014010, China; cyx_imust@163.com (Y.C.); sharkv8088@163.com (Y.S.); ouyangshunli@imust.cn (S.O.); 2 School of Material and Metallurgy, Inner Mongolia University of Science and Technology, Baotou 014010, China

**Keywords:** alumina composites, iron concentrate, bauxite, carbothermal reduction, mechanical properties

## Abstract

Fe-Al_2_O_3_ composites were prepared by pressureless sintering, using Bayan Obo iron concentrate and bauxite as the main raw materials, activated carbon was added as the reducing agent. The effects of different bauxite additions on the phase composition, microstructures, mechanical properties, and the corrosion-resistance were investigated. The results show that the average grain size of alumina decreased as the bauxite content increased. In addition, bauxite contains TiO_2_, CaO, and MgO, that can form a liquid phase at high temperature, causing the heat capacity of the micro-zone around the metal phase to be different, which leads to a change of undercooling and further affects the dislocation density of the metal phase. The plastic deformation ability of the metal phase can be improved with the low dislocation density. Fracture mechanism analysis indicated that the metal phase absorbed energy through plastic deformation. The optimum performance of the sample was as follows: the flexural strength was 310 MPa, the hardness 12.14 GPa, the alkali resistance 98.32%, and the acid resistance 95.44%.

## 1. Introduction

Alumina (Al_2_O_3_) has the advantages of a high melting point, a high level of hardness, excellent heat and corrosion resistance and good electrical insulation. Hence, alumina can be used under extreme conditions. Because of its simple production process and low cost, alumina is one of the most important and widely used materials in structure applications [1,2]; for instance, in cutting tool materials, wear-resistant parts, and bioceramics, as well as in the aerospace, energy, and chemical fields [3,4,5,6]. Therefore, the study of Al_2_O_3_ ceramic material has greatly attracted many researchers and developers in recent decades. However, due to the inherent defect of room temperature brittleness, the application of alumina is greatly restricted [7,8,9,10]. Therefore, the aim of many studies has been to identify the method of toughening alumina ceramics. Around this topic, various toughening mechanisms have been investigated, including whisker toughening, self-toughening, phase transformation toughening, and particle dispersion toughening [11]. Among these, particle toughening has been proved to be a promising toughening method. The cracks can be hindered by the introduction of a ductile metal phase into a brittle ceramic in various ways, including crack passivation, deflection, pinning, and metal ion extraction. In addition, the sintering performance of ceramics can be improved by the introduction of a ductile metal phase [12]. Thus, for decades much attention has been focused on the development of Al_2_O_3_-metal composites. The performance of Al_2_O_3_-metal composites can be improved by introducing various metal particles such as Ni [13], Cu [6], Mo [14], Ti [15], etc. The Ti-Al_2_O_3_ composite was prepared by using pressureless sintering, and the flexural strength was 160 MPa [16]. The flexural strength of Al_2_O_3_-Cr composites prepared by hot-pressing sintering was 349 MPa [17]. Although the properties of the materials are excellent, the second phase of the metal introduced is generally expensive, making the preparation cost of the composite materials higher. Therefore, in this paper, we used metallic iron which is cheap and readily available as the toughening phase of alumina ceramics.

The traditional method for preparing a ceramic composite is mostly using fine chemical powder, which has the disadvantages of high energy consumption and high cost. In recent years, many researches have been conducted on the preparation of ceramic materials from natural minerals, exploring a new way to prepare high performance composites at low cost [18]. Many researchers synthesise composites by using natural ilmenite which is reduced by carbon, aluminum, magnesium, calcium, and other reduction agents to form hard phase TiC, TiN. and metal phase Fe. [19,20]. It will not only reduce the cost but also obtain better properties than synthesizing ceramic composites by reaction sintering from minerals. This is based on the consideration that there is a high grade of iron oxide in the iron ore after magnetic separation which can be used as a source of iron for preparing Fe-Al_2_O_3_ composites. Bauxite is widely distributed in nature as a natural alumina mineral. The high-grade bauxite contains alumina up to 85% or more, and the other components such as SiO_2_, TiO_2,_ and CaO can be used as natural additives for alumina sintering, that can effectively reduce the sintering temperature and porosity and improve the properties of the materials [21]. At present, bauxite is only used as a raw material for extracting alumina or preparing refractories [22,23,24,25,26]. Therefore, the part of pure alumina is replaced by bauxite to prepare Fe-Al_2_O_3_ composites in this paper in order to further reduce the production cost of the composite materials. Moreover, it is also possible to explore a way to produce high-performance ceramic composites at low cost using natural minerals. For minerals, high-performance composites can be prepared by taking advantage of their natural properties without physical or chemical separation.

In this paper, iron concentrate and bauxite were used as main raw materials to prepare Fe-Al_2_O_3_ composites in a pressureless sintering furnace by the carbothermal reduction method. The effects of different bauxite contents on the phase composition, microstructures, mechanical properties, and the corrosion-resistant were investigated.

## 2. Materials and Methods

### 2.1. Materials

Bayan Obo iron concentrate (provided by the team studying mineral processing, Inner Mongolia University of Science and Technology, Baotou, China), bauxite (provided by Mantanghong Co., Gongyi, China), Al_2_O_3_ powder (99.9% of purity, 2 μm of average particle size, produced by Shanghai Macklin Biochemical Co., Ltd., Shanghai, China) and activated carbon (99% of purity, 0.5–1 μm average particle size, produced by Tianjin Damao Chemical Reagent Co., Ltd., Shanghai, China) were used as the raw materials. The chemical composition of the raw materials are shown in Table 1.

**Figure 1:**
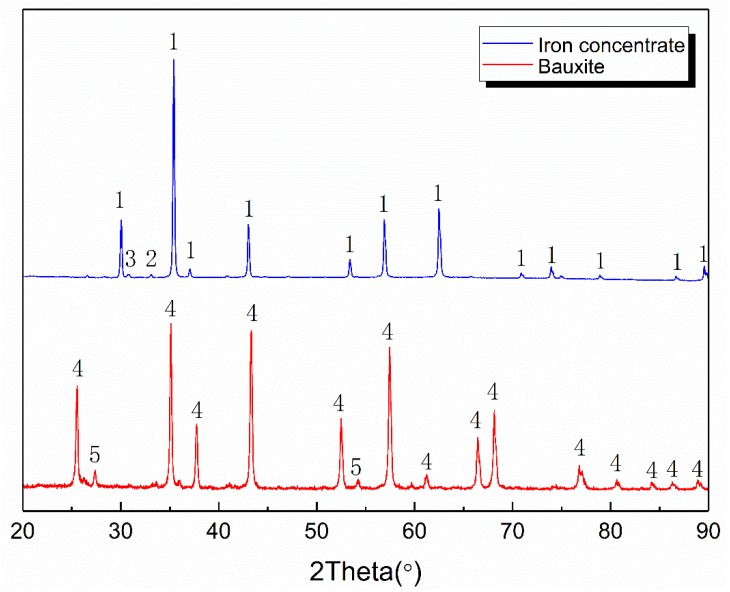
X-ray diffraction patterns of iron concentrate and bauxite (1-Fe_3_O_4_, 2-Fe_2_O_3_, 3-Diopside, 4-Al_2_O_3_, 5-TiO_2_).

**Figure 2:**
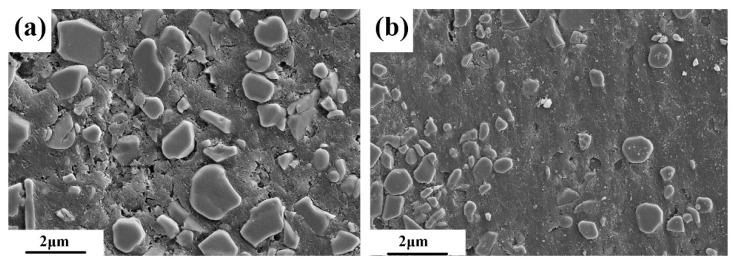
Scanning Electron Microscope (SEM) images of alumina (**a**) and bauxite (**b**).

The X-ray diffraction (XRD) patterns of the Bayan Obo iron concentrate powder and bauxite are shown in Figure 1. As shown, the iron concentrate mainly consists of magnetite, hematite, and a small amount of diopside. Bauxite mainly contains alumina and titanium dioxide. After sintering, the magnetite and hematite are transformed into iron by carbothermal reduction. Figure 2 shows the Scanning Electron Microscope (SEM) images of alumina and bauxite.

### 2.2. Methods

A batch of composite was prepared according to Table 2. According to the content of iron concentrate, additionally required reducing agent is added. The powder was mixed for 4 h in a ball mill at 300 rpm; the ball milling medium was anhydrous ethanol and the ball-to-powder radio was 4:1. Slurry was obtained and then dried for 24 h at 95 °C. Samples of φ 40 mm × 5 mm were hydraulically compacted by uniaxial pressing at 35 MPa. The samples were sintered under graphite powder in order to prevent the oxidation of iron. The shaped samples were fired at temperatures of 1380 °C in a pressureless sintering furnace. The heating rate was 4 °C/min under a holding time of 180 min.

The crystalline phases were determined by XRD (X’pert Pro Powder, PANalytical, Almelo, The Netherlands). A copper target was used, and the X-ray tube was operated at 30 KV and 40 mA, with a 2θ scan range from 20° to 90°, a step size of 0.02° and a scan speed of 0.3 s/step. The microscopic structures were investigated by SEM (SUPRA 55 FESEM, Carl Zeiss, Jena, Germany), equipped with an Oxford EDS and EBSD analysis system.

The sample surfaces were polished in a colloidal silica suspension for 1 h to yield a stress-free surface for high-quality electron backscatter diffraction (EBSD) images. The EBSD images were taken at a step size of 100 nm and the samples were detected at a tilt of 70° to the beam.

The densities were measured using the Archimedes method. Each value used is the mean value of measurements from five samples. Linear shrinkage (*LS*) was determined using the length difference between the green (*L*_1_) and the fired sample (*L*_2_), and calculated as shown in Equation (1):

(1) $$LS\left(\%\right)=100\times \left(L_{1}-L_{2}\right)/L_{1}$$

The hardness of the samples was measured by the Vickers hardness method. The three-point flexural strength (*FS*) of the rectified parallelepiped bars (3 mm × 4 mm × 40 mm) of the sample was tested using the CSS-88000 electronic universal testing machine (Changchun testing machine research institute, Changchun, China), and then calculated as shown in Equation (2):

(2) $$FS\left(\mathrm{MPa}\right)=3\times F\times l/\left(2\times b\times h^{2}\right)$$

where *F* is the breaking load (N), *l* is the span between the support rods (mm), *b* is the width of the test sample (mm) and *h* is the minimum thickness of the test sample measured after the test along the broken edge (mm).

Corrosion experiments were carried out on sample particles with a particle size of 0.5 to 1.0 mm using a mass fraction of 20% NaOH and 20% H_2_SO_4_. The temperature was 100 °C and the corrosion time was 1 h, calculated as shown in Equation (3):

(3) $$K=\left(m_{1}/m\right)\times 100\%$$

where *K* is the acid (alkali) resistance, $m_{1}$ is mass after corrosion, and m is mass before corrosion.

## 3. Results and Discussion

### 3.1. Mechanical Properties

The mechanical properties and corrosion-resistance of four samples were measured, and the results are given in Table 3. The density is about 4.0 g/cm^3^ and the linear shrinkage is about 17.5%. There is no significant difference between the four samples in density and linear shrinkage. The flexural strength of sample B1 was 240 MPa, and that of B2 was 250 MPa, the increase was not obvious. The flexural strength of B3 reached 310 MPa, while that of sample B4 decreased slightly to 290 MPa. The trend of hardness and flexural strength was basically the same, and the B3 sample reached the maximum value, with a slight variation trend of the four samples. There was no significant change in acid and alkali resistance, the alkali resistance was about 98.3%, and the acid resistance about 95.5%.

It is worth noting that the content of iron concentrate and reducing agent in the four samples is the same, while the content of bauxite is different. However, the mechanical properties of the four samples were significantly different. Hence, the detection of phase and microstructure was carried out to analyze the main reasons for the improvement of the mechanical properties of bauxite.

### 3.2. Phases and Microstructures

**Figure 3:**
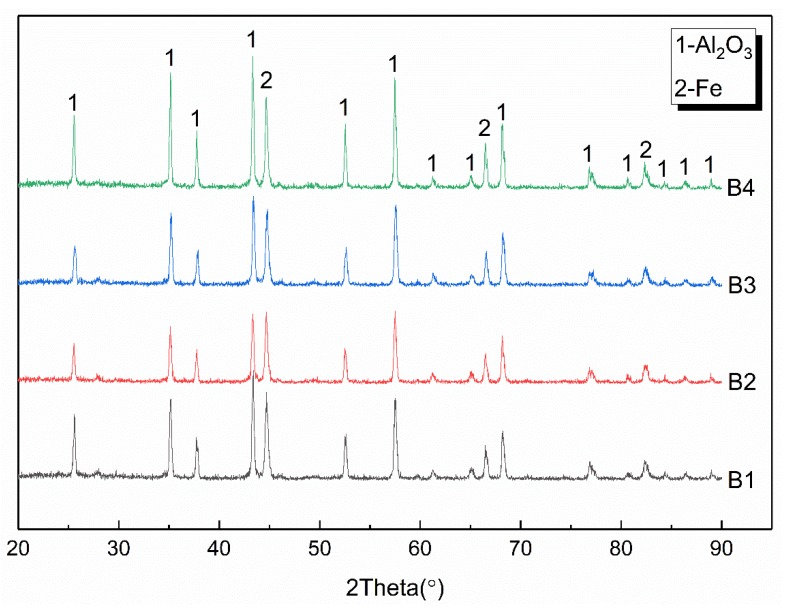
X-ray diffraction patterns of the samples B1–B4.

In order to determine the phase composition of composites, XRD analysis was carried out on the samples. Figure 3 shows the XRD patterns of samples sintered at 1380 °C with different bauxite additions. Samples B1, B2, B3, and B4 are displayed in order from bottom to top. As shown in the figure, diffraction peaks of only Al_2_O_3_ and Fe were found in all four samples. This indicated that the reduction reaction can be carried out completely at a sintering temperature of 1380 °C. In addition, no diffraction peaks related to TiO_2_ which is contained in the bauxite were found in the XRD patterns. The reason for this phenomenon is that TiO_2_ combined with the oxides such as SiO_2_, CaO to form a liquid phase at high temperature. During the cooling process, this liquid phase solidifies and forms a glass phase. The oxides such as SiO_2_, CaO, and TiO_2_ in raw minerals are not harmful substances in the process of materials preparation. On the contrary, they can be used as good sintering aids to effectively promote the sintering of the composites, reduce the sintering temperature, and reduce the porosity [7].

**Figure 4:**
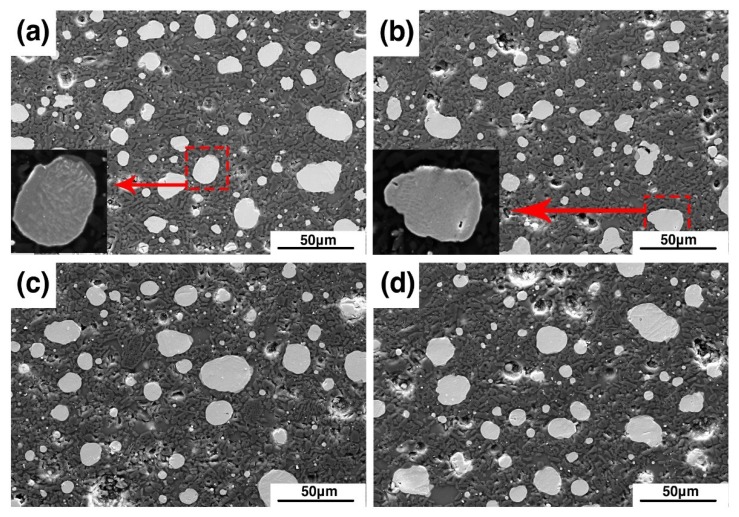
SEM images of samples B1 (**a**), B2 (**b**), B3 (**c**), and B4 (**d**).

Figure 4 shows the microstructure of samples B1–B4 after sintering at 1380 °C. The white particles are metallic iron, and the black region consists of alumina matrix and glass phases. The iron particles in the four samples are circular or oval. The reason for this phenomenon is that the carbon content in the sample is excessive, and the iron carburizes with the remaining carbon. As the amount of carburization increases, the melting point of the Fe-C alloy gradually decreases. At the same sintering temperature, the lower the melting point, the lower is the viscosity of the liquid phase. Thus, the metal particles in the sample gradually became circular. 

Through observation of the microstructure, it was found that there are two issues worth paying attention to. First, it was shown that the grain size of alumina declined with the addition of bauxite. Second, the existence of grain boundaries was found in iron particles. This may be the reason why the mechanical properties can be improved by the addition of bauxite in the samples.

**Figure 5:**
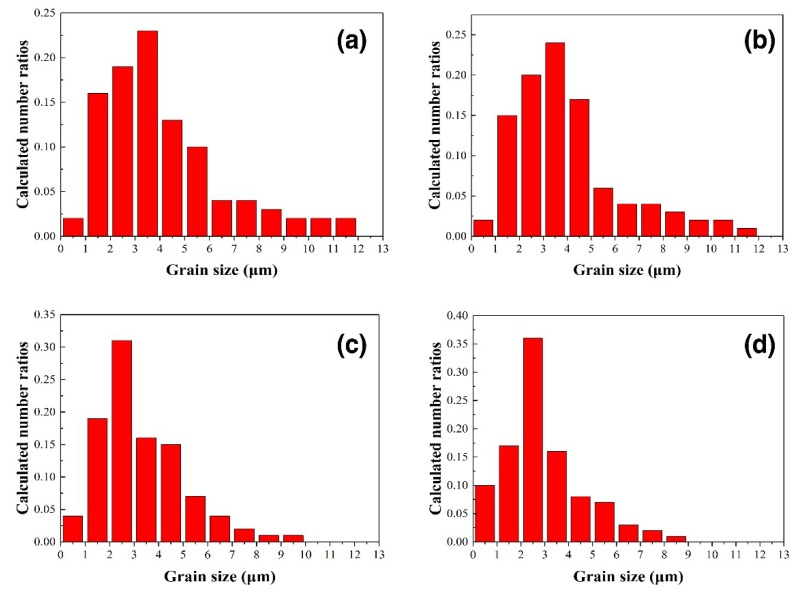
Alumina grain size distribution map of samples B1 (**a**), B2 (**b**), B3 (**c**), and B4 (**d**).

**Figure 6:**
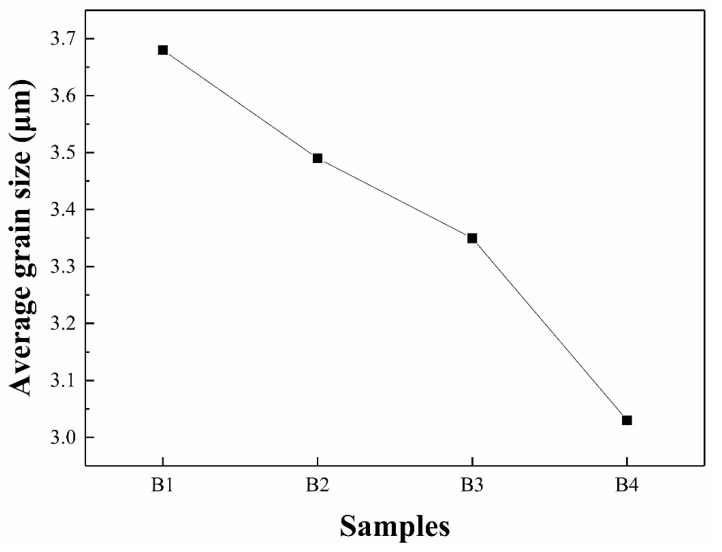
Average size of alumina grains in samples B1–B4.

Alumina is derived from pure alumina and bauxite, and the alumina particles in the bauxite are finer. Therefore, with the increase of bauxite content, the small size alumina grains in the sample gradually increased. The alumina grain size of the four samples was statistically analyzed to quantitatively analyze the size of the alumina grains. Figure 5a–d shows the alumina grain size distribution of samples B1, B2, B3, and B4, respectively. In sample B1, the grain size is mainly between 1.5 μm and 5.5 μm, and the grain proportion of 3.5 μm was the largest. Compared with sample B1, sample B2 has a reduced content of large-size grains. The alumina grains of sample B3 were mainly distributed between 1.5 μm and 4.5 μm, with the highest proportion of 2.5 μm. The ratio of large-size alumina grain size of sample B4 was even lower, and that of 2.5 μm size is about 35%. Figure 6 shows the average size of alumina grains in the four samples. With the increase of bauxite, the average particle size of alumina decreased from 3.7 μm to 3.0 μm. On the whole, the grain size of sample B3 is finer and the size distribution is more uniform, which is one of the reasons for the higher flexural strength of sample B3.

### 3.3. EBSD Analysis

**Figure 7:**
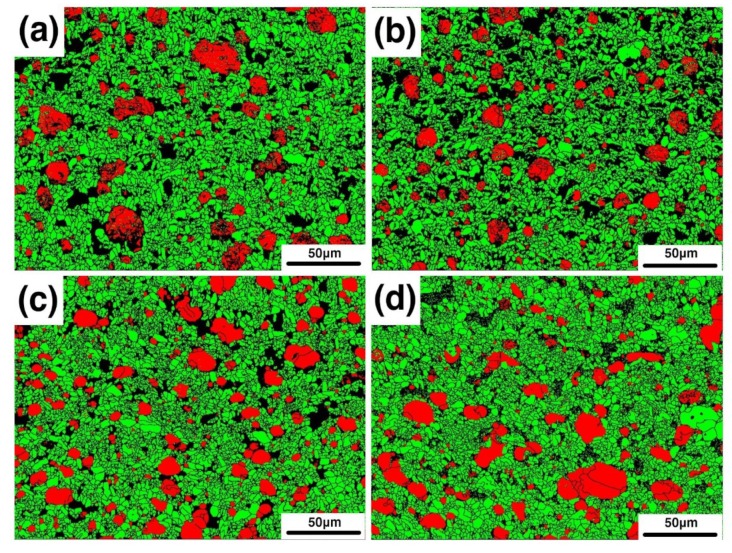
Phase distribution of samples B1 (**a**), B2 (**b**), B3 (**c**), and B4 (**d**).

**Figure 8:**
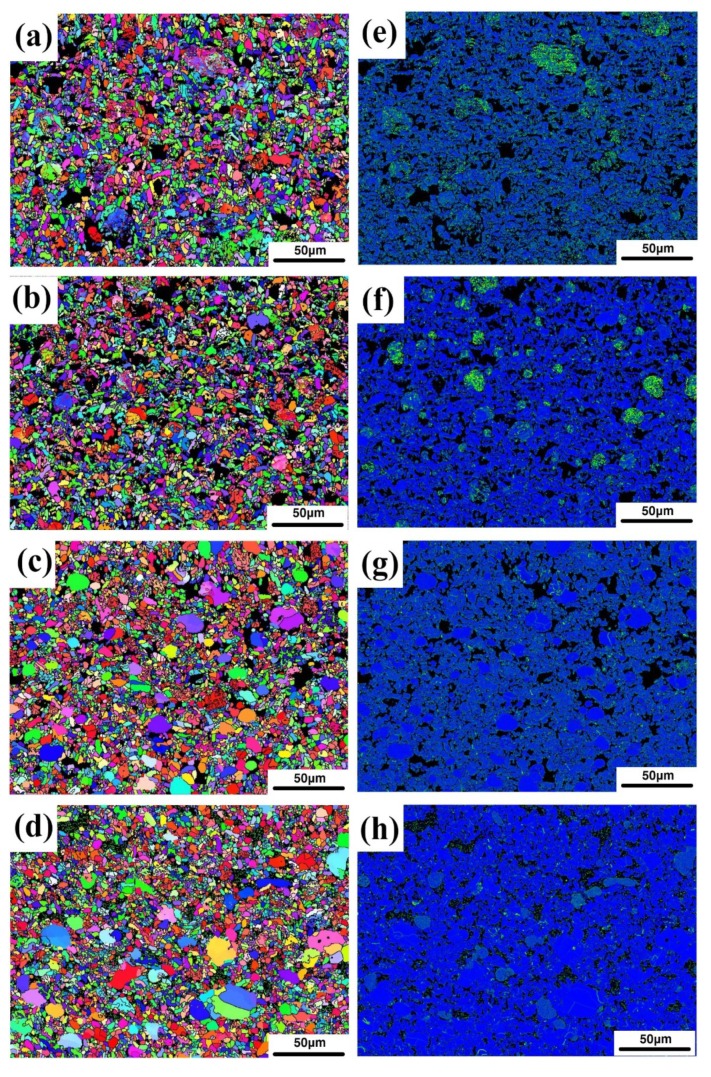
The Inverse Pole Figure X-axis (IPFX) distribution of samples B1 (**a**), B2 (**b**), B3 (**c**), B4 (**d**) and stress distribution of samples B1 (**e**), B2 (**f**), B3 (**g**), B4 (**h**).

In order to analyze the second issue mentioned in the previous section, namely the grain boundaries of iron particles, EBSD analysis was performed. Figure 7a–d shows the phase distribution of samples B1, B2, B3, and B4. The red particles are the metal phase, the green crystals are the alumina phase, and the black regions are the glass phase and the uncalibrated areas. As can be seen in the figure, there are indeed a large number of grain boundaries in the iron particles. It was also found that as the amount of bauxite addition increased, the number of grain boundaries gradually decreased. The iron particles gradually changed to a single crystal or consisted of several subgrains. Figure 8a–d shows Inverse Pole Figure X-axis (IPFX) diagrams of samples B1, B2, B3, and B4, respectively, while the Figure 8e–h shows the stress distribution of the samples B1–B4. It can be seen that the orientation of the iron particles in sample B1 is disordered and random, with a large number of grain boundaries, and the stress of the iron particles is large. It was found that the grain boundary of the iron particles depends on the stress. The grain boundaries of the iron particles with high stress are numerous, and the orientation of the iron particles is also disordered. However, iron particles with small stress have no grain boundary and their orientation is uniform. With the bauxite addition increase, the orientation of the iron particles gradually becomes uniform. It is inferred that the cause of stress and grain boundaries is due to the glass phase in the sample. To confirm this point, we first analyzed the composition of the glass phase.

**Figure 9:**
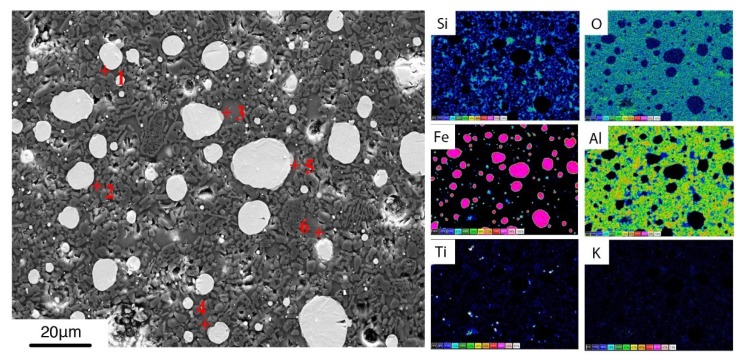
Energy Dispersive Spectrometer (EDS) analyze of sample B3.

According to the chemical composition of bauxite, it contains a large amount of Al_2_O_3_. In addition, there are other oxides such as TiO_2_, SiO_2,_ and CaO contained in the bauxite. Since there was no diffraction peak associated with TiO_2_ in the XRD detection, it was inferred that these oxides formed a liquid phase at high temperature and formed a glass phase in the cooling process. Therefore, with the increase of bauxite addition, the content of TiO_2_, SiO_2,_ and CaO also increases, and more liquid phase is produced in the micro-area environment of the metal droplets. Taking B3 sample as an example, as shown in Figure 9, EDS was performed on the sample and “Quantmap” function was used to obtain a quantitative element surface distribution map. The chemical composition at different points of the glass phase was detemined and averaged, the results are listed in Table 4.

According to the literature [28], the heat capacity of silicate melt is calculated by using Equation (4):

(4) $$C_{p}=\underset{i}{\sum }X_{i}C_{P,j}$$

where $X_{i}$ is the mole fraction, $C_{P,j}$ is the partial molar heat capacity of the components of a solution.

By calculation, the heat capacity of the glass phase in sample B3 is 139.6 J/(mol·K) when melting at 1380 °C while the heat capacity of the alumina crystal is about 130 J/(mol·K) at 1380 °C [29]. On the one hand, the heat capacity of the silicate liquid phase is larger than that of alumina. On the other hand, heat will be released during the solidification of the silicate liquid phase. Hence, more silicate liquid phases provide additional heat during the cooling process of the iron droplets, resulting in a decrease in the cooling rate, which causes the iron droplets to be decreased on undercooling. From sample B1 to B4, as the amount of silicate liquid phase increases, the cooling rate gradually decreases, resulting in a gradual decrease in the undercooling of the iron droplets.

**Figure 10:**
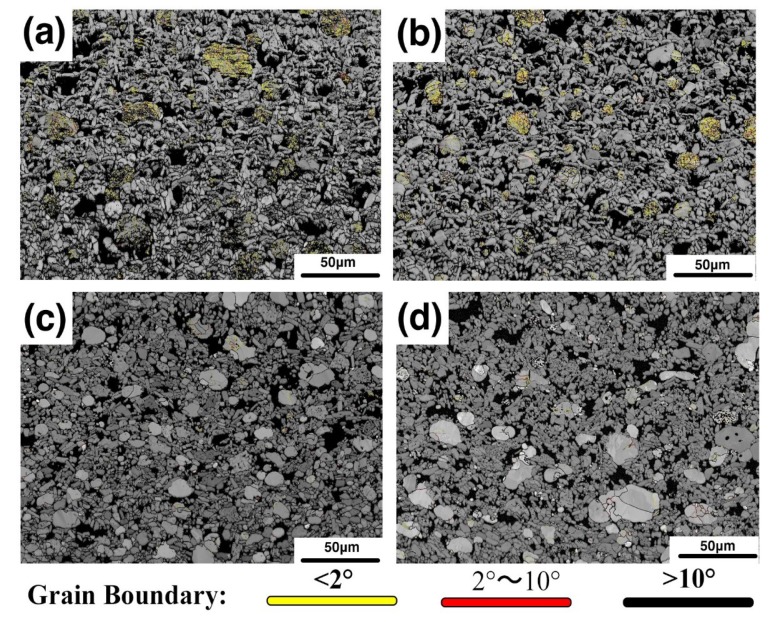
The grain boundary distribution of samples distribution of samples B1 (**a**), B2 (**b**), B3 (**c**), and B4 (**d**).

To investigate the cause of the grain boundary formation, the grain boundaries inside the iron particles are classified according to angles, wherein 2° is represented by yellow, 2°–10° is represented by red, and greater than 10° is represented by black. As shown in Figure 10 it was found that in the samples B1 and B2, most of the grain boundaries were yellow, that is, they were all low-angle grain boundaries, and only a very small number of high-angle grain boundaries.

In sample B1, the cooling rate of the metal droplets is relatively fast due to the smaller heat capacity of the micro-area environment around the metal droplet. It is well known that at large cooling rates, thermal stress caused by temperature gradients induces the occurrence of dislocations. This conclusion is consistent with many published studies [30,31,32]. According to the dislocation model [33], the low-angle grain boundary can be regarded as consisting of a large number of dislocations. Therefore, the stress of the iron particles in the sample is mainly due to the thermal stress caused by the undercooling, and the larger stress causes an increase in dislocations in the iron particles. On the basis of the solidification principle, a large number of dislocations cause hardening of the metal, thereby affecting its plastic deformation. In this study, the low-angle grain boundary weakens the reinforcing effect of the metal phase in the composite material.

**Figure 11:**
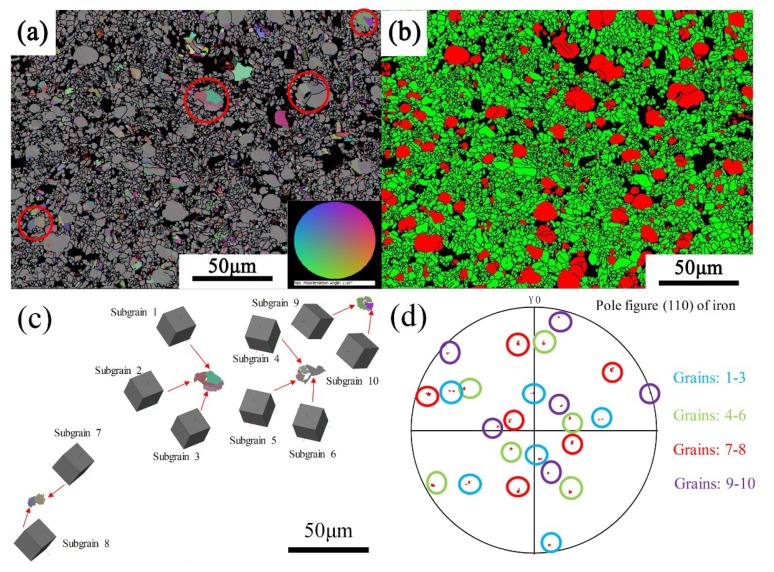
The orientation relationship of subgrains in sample B3. (**a**) low-angle grain boundaries distribution; (**b**) phase distribution; (**c**) orientation relationship of subgrains; (**d**) subgrains plotted in (110).

In addition, although the thermal stress was significantly reduced in the B3 and B4 samples, it can be seen from Figure 9 that a number of low-angle grain boundaries are still present inside the iron particles. Moreover, the iron particles marked by a red circle in Figure 11a contain two or more subgrains. It is inferred that this is caused by nucleation of the grain boundary which was in the metal phase. In order to prove the conclusion, the orientation of these crystals was analyzed, as shown in Figure 11c. The results indicated that the subgrains contained in each iron particle have the same orientation. To further prove this conclusion, the corresponding crystal planes were plotted in stereographic projections, as shown in Figure 11d. The marked planes indicate the parallel planes between the subgrains.

**Figure 12:**
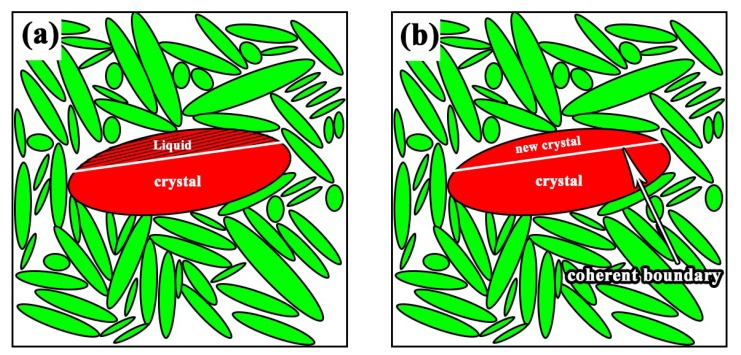
Schematic illustration of grain boundary nucleation. (**a**) Nucleation period; (**b**) Crystal growth period.

Since the crystal lattice of the core and parent crystal match perfectly, the interface between the core and the crystal is partially or completely coherent, so that the nucleation energy can be further reduced [34,35]. Figure 12 is the schematic diagram of grain boundary nucleation. The “crystal” in Figure 12a is a crystal that has grown, and its lower surface is in contact with the ceramic phase, and exhibits a round crown interface due to poor wettability. The interface between the newly formed core on the upper surface and the crystal is coherent. Thus, the shape of the surface is straight, which is a low-energy interface. With time, atoms in the liquid phase continue to accumulate at the interface, the crystals keep growing and gradually form new crystals named “new crystal”, as shown in Figure 12b. Hence, an iron particle is composed of two or more crystals with the same orientation, and all the grain boundaries are straight.

Because of the different liquid content in the sample, the cooling speed of the iron particles in the micro-area is different, resulting in different undercooling of the iron particles. The content of bauxite in the sample has a great influence on the thermal stress of the metal droplets during solidification. There are a large number of low-angle grain boundaries in sample B1, which affect the plastic deformation during the loading process. In sample B3 there are only a few low-angle grain boundaries. During the loading of the sample, more energy is absorbed by the iron particles through obvious plastic deformation. This is another important reason for the excellent mechanical properties of sample B3.

### 3.4. Fracture Mechanism

**Figure 13:**
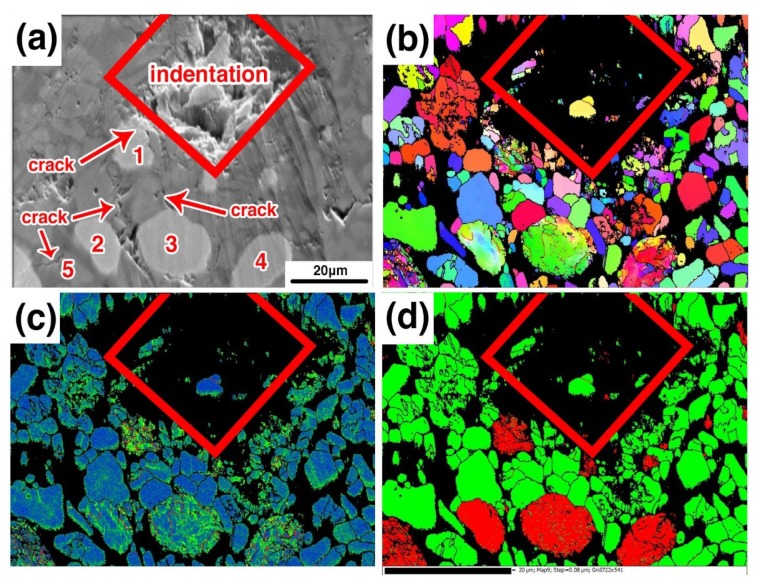
Crack propagation morphology of sample B3; (**a**) SEM image, (**b**) IPFX image, (**c**) stress distribution, (**d**) phase distribution.

To study whether the metal phase is plastically deformed during the stress process, the EBSD of the crack propagation was analyzed in sample B3. Figure 13a is the SEM image of the crack propagation. It was found that cracks are generated on both the left and right sides of the indentation. Figure 13b is the IPFX image, and Figure 13c shows the stress distribution. It can be seen in the figure that there is a large orientation transformation in iron particles 1#–4#, and the stress of the iron particles is increased. It confirms that the iron particles have obvious plastic deformation and a large number of dislocations in the interior under the action of external force. The closer the metal particles get to the indentation, the greater the orientation transformation and the more are the dislocations generated. Around the iron particles, the alumina grains next to the iron particles still maintain their integrity, while the alumina grains, such as alumina grain 5#, which is far away from the iron particles, the fracture mechanism of this grain is transgranular fracture. It is indicated that the metal phase in the composites improves the mechanical properties of the materials in two ways, one is the deflection of the crack, and the other is the absorption of energy by plastic deformation.

## 4. Conclusions

(1) Fe-Al_2_O_3_ composites were successfully prepared by using pressureless sintering from iron concentrate and bauxite. The optimum performance of the sample was as follows: the flexural strength was 310 MPa, the hardness 12.14 GPa, the alkali resistance 98.32%, and the acid resistance 95.44%. The composites have excellent mechanical properties, while have low production cost and a simple production process. Therefore, the composites have broad application prospects.

(2) Since the alumina crystals of bauxite are finer than that of pure alumina, the average size of the alumina grains in samples with high bauxite content is finer.

(3) With the increase of bauxite addition, the silicate liquid phase exhibits a growing trend. Since the heat capacity of the silicate liquid phase is greater than that of the alumina crystal, the undercooling of the iron droplets gradually decreases as the bauxite increases. In the samples with low undercooling, there is no large amount of low-angle grain boundaries in the metal phase due to the lower thermal stress, and the metal phase absorbs energy by plastic deformation to enhance the strength of the composite during the process of the sample loading.

## Figures and Tables

**Table 1:** The chemical composition of the raw materials.

Name	TFe	FeO	Al_2_O_3_	SiO_2_	CaO	MgO	TiO_2_	Na_2_O	MnO_2_	S
Iron concentrate (%)	65.99	27.4	-	2.16	1.00	0.68	-	0.15	0.8	0.77
Bauxite (%)	1.84	-	87.04	5.01	0.23	0.21	4.12	-	-	-
Al_2_O_3_ (%)	0.1	-	99.5	0.2	-	-	0.1	0.1	-	-

**Table 2:** The composition of composite formulation (wt%).

No.	Iron Concentrate	Bauxite	Alumina
B1	50	10	40
B2	50	15	35
B3	50	20	30
B4	50	25	25

**Table 3:** Mechanical properties and corrosion-resistance of samples B1~B4.

No.	Density (g/cm^3^)	Linear Shrinkage (%)	Flexural Strength (MPa)	Hardness (GPa)	Alkali-Resistance (%)	Acid-Resistance (%)
B1	3.98	17.32	240	11.47	98.25	95.51
B2	3.98	17.27	250	11.62	98.30	95.47
B3	4.02	17.55	310	12.14	98.32	95.44
B4	4.01	17.49	290	11.97	98.29	95.52

**Table 4:** Chemical composition of the glass phase in sample B3 and the partial molar heat capacity at 0.1 MPa/mol%.

Spectra	SiO_2_	Fe_2_O_3_	Al_2_O_3_	TiO_2_	MgO	CaO	K_2_O
Point 1	39.2	24.6	24.1	6.3	1.9	2.9	1.0
Point 2	40.7	26.5	23.5	5.9	1.1	1.1	1.2
Point 3	40.2	26.4	24.5	5.5	1.2	1.0	1.2
Point 4	37.7	25.3	25.2	6.0	2.5	2.1	1.2
Point 5	39.1	25.6	24.1	4.0	2.0	2.9	2.2
Point 6	39.9	25.0	23.9	4.9	2.2	2.2	1.9
Average content	39.5	25.6	24.2	5.4	1.8	2.0	1.5
$C_{P,j}$, J/(mol·K) [27]	80.0	229.0	157.6	111.8	99.7	99.9	97.0

## References

[1] Xu H.Q., Wang Z., Wu J.Y., Li Q.G., Liu M.J., Li Y.Y. (2016). Mechanical properties and microstructure of Ti/Al_2_O_3_ composites with Pr_6_O_11_ addition by hot pressing sintering. Mater. Des..

[2] Yu M.X., Zhang J.X., Li X.G., Liang H.Q., Zhong H., Duan Y.S., Jiang D.L., Liu X.J., Huang Z.G. (2015). Optimization of the tape casting process for development of high performance alumina ceramics. Ceram. Int..

[3] Marmier A., Lozovoi A., Finnis M.W. (2003). The α-alumina (0001) surface: Relaxations and dynamics from shell model and density functional theory. J. Eur. Ceram. Soc..

[4] Hirvikorpi T., Vähä-Nissi M., Nikkola J., Harlin A., Karppinen M. (2011). Thin Al_2_O_3_ barrier coatings onto temperature-sensitive packaging materials by atomic layer deposition. Surf. Coat. Technol..

[5] Broniszewski K., Wozniak J., Kostecki M., Czechowski K., Jaworska L., Olszyna A. (2015). Al_2_O_3_-V cutting tools for machining hardened stainless steel. Ceram. Int..

[6] Dehm G., Scheu C., Rühle M., Raj R. (1998). Growth and structure of internal Cu/Al_2_O_3_ and Cu/Ti/Al_2_O_3_ interfaces. Acta Mater..

[7] Sun J.L., Liu C.X., Zhang X.H., Wang B.W., Ni X.Y. (2009). Effect of diopside addition on sintering and mechanical properties of alumina. Ceram. Int..

[8] Zhang X.F., Li Y.C. (2010). On the comparison of the ballistic performance of 10% zirconia toughened alumina and 95% alumina ceramic target. Mater. Des..

[9] Pillai S.K.C., Baron B., Pomeroy M.J., Hampshire S. (2004). Effect of oxide dopants on densification, microstructure and mechanical properties of alumina-silicon carbide nanocomposite ceramics prepared by pressureless sintering. J. Eur. Ceram. Soc..

[10] Zhang W., Smith J.R., Evans A.G. (2002). The connection between ab initio calculations and interface adhesion measurements on metal/oxide system: Ni/Al_2_O_3_ and Cu/Al_2_O_3_. Acta Mater..

[11] Pettersson P., Johnesson M. (2003). Thermal shock properties of alumina reinforce with Ti (C, N) whiskers. J. Eur. Ceram. Soc..

[12] Cannon R.M., Korn D., Elssner G., Ruhle M. (2002). Fracture properties of interfacially doped Nb-Al_2_O_3_ bicrystals: Ⅱ, relation of interfacial bonding, chemistry and local plasticity. Acta Mater..

[13] Irshad H.M., Hakeem A.S., Ahmed B.A., Ali S., Ehsan M.A., Laoui T. (2018). Effect of Ni content and Al_2_O_3_ particle size on the thermal and mechanical properties of Al_2_O_3_/Ni composites prepared by spark plasma sintering. Int. J. Refract. Metals Hard Mater..

[14] Shon I.J. (2018). Rapid consolidation of nanostructured Mo-Al_2_O_3_ composite from mechanically synthesized powders. Ceram. Int..

[15] Wu C., Li Y.K., Wang Z. (2016). Evolution and mechanism of crack propagation method of interface in laminated Ti/Al_2_O_3_ composite. J. Alloy Compd..

[16] Hu H.Q., Wang Z., Wu J.Y., Xu X.J. (2017). Effects of Pr_6_O_11_ on the microstructure and mechanical properties of Ti/Al_2_O_3_ composites prepared by pressureless sintering. Ceram. Int..

[17] Marchin C., Katarzyna P. (2007). Processing; microstructure and mechanical properties of Al_2_O_3_-Cr nanocomposites. J. Eur. Ceram. Soc..

[18] Burden S.J., Hong J., Rue T.W. (1988). Comparison of hot-Isostatically pressed and uniaxially hot-pressed alumina-titanium carbide cutting tools. Am. Ceram. Soc. Bull..

[19] Khoshhal R., Soltanieh M., Boutorabi M.A. (2015). The effect of Fe_2_Al_5_ as reducing agent in intermediate steps of Al_2_O_3_/TiC-Fe composite production process. Int. J. Refract. Metals Hard Mater..

[20] Khoshhal R., Soltanieh M., Boutorabi M.A. (2015). Investigation on the reactions sequence between synthesized ilmenite and aluminum. J. Alloy Compd..

[21] Ai X.B., Li Y., Gu X.M., Cang D.Q. (2013). Development of ceramic based on steel slag with different magnesium content. Adv. Appl. Ceram..

[22] Li B.W., Deng L.B., Zhang X.F., Jia X.L. (2013). Structure and performance of glass-ceramics obtained by Bayan Obo tailing and fly ash. J. Non-Cryst. Solids..

[23] Chen J.W., Zhao H.Z., Yu J., Zhang H., Li Z.K., Zhang J.Q. (2018). Synthesis and characterization of reaction-bonded calcium alumino-titanate-bauxite-SiC composite refractories in a reducing atmosphere. Ceram. Int..

[24] Maldhure A.V., Tripathi H.S., Ghosh A. (2015). Mechanical properties of mullite-corundum composites prepared from bauxite. Int. J. Appl. Ceram. Technol..

[25] Zhang H.J., Han B., Liu Z.J. (2006). Preparation and oxidation of bauxite-based β-Sialon-bonded SiC composite. Mater. Res. Bull..

[26] Ren B., Sang S.B., Li Y.W., Xu Y.B. (2015). Effects of oxidation of SiC aggregates on the microstructure and properties of bauxite-SiC composite refractories. Ceram. Int..

[27] Lange R.A., Navrotsky A. (1992). Heat capacities of Fe_2_O_3_-bearing silicate liquids. Contrib. Mineral. Petr..

[28] Stebbins J.F., Carmichael I.S.E., Moret L.K. (1984). Heat capacities and entropies of silicate liquids and glasses. Contrib. Mineral. Petr..

[29] Barin I. (1995). Thermochemical Data of Pure Substances.

[30] Nakano S., Chen X.J., Gao B., Kakimoto K. (2011). Numerical analysis of cooling rate dependence on dislocation density in multicrystalline silicon for solar cells. J. Cryst. Growth..

[31] Gao B., Kakimoto K. (2014). Three-dimensional analysis of dislocation multiplication in single-crystal silicon under accurate control of cooling history of temperature. J. Cryst. Growth..

[32] Gao B., Nakano S., Harada H., Miyamura Y., Kakimoto K. (2013). Effect of cooling rate on the activation of slip systems in seed cast-grown monocrystalline silicon in the [1] and [111] directions. Cryst. Growth Des..

[33] Mullin J.W. (2001). Crystallization.

[34] Froseth A.G., Derlet P.M., Swygenhoven H.V. (2004). Dislocations emitted from nanocrystalline grain boundaries: Nucleation and splitting distance. Acta Mater..

[35] Sangid M.D., Ezaz T., Sehitoglu H., Robertson I.M. (2011). Energy of slip transmission and nucleation at grain boundaries. Acta Mater..

